# Effects of Heavy-Resistance Strength and Balance Training on Unilateral and Bilateral Leg Strength Performance in Old Adults

**DOI:** 10.1371/journal.pone.0118535

**Published:** 2015-02-19

**Authors:** Rainer Beurskens, Albert Gollhofer, Thomas Muehlbauer, Marco Cardinale, Urs Granacher

**Affiliations:** 1 University of Potsdam, Division of Training and Movement Sciences, Research Focus Cognition Sciences, Potsdam, Germany; 2 Albert-Ludwigs-University of Freiburg, Institute of Sport and Sport Science, Freiburg, Germany; 3 ASPIRE—Academy for Sports Excellence, Doha, Qatar; 4 University College London, Institute of Sports Exercise and Health, London, United Kingdom; University of Ottawa, CANADA

## Abstract

The term “bilateral deficit” (BLD) has been used to describe a reduction in performance during bilateral contractions when compared to the sum of identical unilateral contractions. In old age, maximal isometric force production (MIF) decreases and BLD increases indicating the need for training interventions to mitigate this impact in seniors. In a cross-sectional approach, we examined age-related differences in MIF and BLD in young (age: 20–30 years) and old adults (age: >65 years). In addition, a randomized-controlled trial was conducted to investigate training-specific effects of resistance vs. balance training on MIF and BLD of the leg extensors in old adults. Subjects were randomly assigned to resistance training (n = 19), balance training (n = 14), or a control group (n = 20). Bilateral heavy-resistance training for the lower extremities was performed for 13 weeks (3 × / week) at 80% of the one repetition maximum. Balance training was conducted using predominately unilateral exercises on wobble boards, soft mats, and uneven surfaces for the same duration. Pre- and post-tests included uni- and bilateral measurements of maximal isometric leg extension force. At baseline, young subjects outperformed older adults in uni- and bilateral MIF (all p < .001; *d* = 2.61–3.37) and in measures of BLD (p < .001; *d* = 2.04). We also found significant increases in uni- and bilateral MIF after resistance training (all *p* < .001, *d* = 1.8-5.7) and balance training (all *p* < .05, *d* = 1.3-3.2). In addition, BLD decreased following resistance (p < .001, *d* = 3.4) and balance training (p < .001, *d* = 2.6). It can be concluded that both training regimens resulted in increased MIF and decreased BLD of the leg extensors (HRT-group more than BAL-group), almost reaching the levels of young adults.

## Introduction

Bilateral deficit (BLD) is a well-known neurophysiological phenomenon characterized by a reduction in force generating capacity during synchronous bilateral contractions when compared to the sum of identical unilateral contractions [1–4]. Since many everyday activities (e.g., rising from a chair) require simultaneous bilateral contractions, this well-known neuromuscular phenomenon has received a lot of attention during the past decades [3,5,6]. A large number of studies focused on BLD and maximal voluntary force production (cf. [7] for a review) and found BLDs between 5–25%, depending on the applied test methodology (i.e., isometric vs. dynamic) [6], the investigated population (e.g., young, middle-aged, old adults) [8], and muscle groups (i.e., lower vs. upper extremities [3]).

The BLD is attributed to modifications in neuromuscular and cortical control during uni- and bilateral homonymous muscle contractions [9]. Different theories have been proposed to explain BLD. Some of the causes seem to be: (1) inhibited spinal reflexes (i.e., sensory activity from one limb inhibits motor neuron activation in the contralateral limb and vice versa) [10], (2) a reduction in motor neuron excitability of particularly fast-twitch motor neurons during bilateral muscle contractions [11], and (3) cortical inhibition related to the interaction of the primary motor cortex of the two hemispheres when performing bilateral contractions [12]. In particular, cortical inhibition seems to be a prominent mechanism during bilateral exertions, where the motor cortex of the contralateral hemisphere inhibits the ipsilateral motor cortex via transcallosal pathways [13]. Cortical inhibition decreases neural drive to the activated muscles during bilateral contractions, thereby resulting in force decrements [10,13]. In this context, BLD reflects a neural inhibitory mechanism during symmetrical bilateral muscle contractions [4,11]. Thus, it can be assessed as a proxy for the role of central activation during force generation.

It is well established that aging reduces maximal voluntary force production and muscular power [6,14]. Still, there is controversy regarding age-related effects on BLD during maximal voluntary force production. Some studies reported no age-differences in BLD [6,8], whereas others observed small [15] or large [16] deficits in old compared to young adults. During isokinetic knee flexion and extension at 45 deg/s, no differences in BLD were found between young (24%), middle-aged (25%), and old (32%) adults [8], although tendencies towards higher BLDs were noticed in seniors. The latter is supported by a study conducted by Vieluf and colleagues [16] who observed increased BLDs in submaximal hand force modulation tasks with advancing age. Young adults showed a BLD of 22%, while old adults reached 34%, indicating that old adults’ capability to activate muscles is particularly impaired during bilateral muscle contractions.

Despite these age-related differences in BLD, studies on the effects of training on BLD are scarce. It is indicated that bilateral resistance training results in reduced BLDs in older women [17]. In contrast, unilateral resistance training resulted in improved uni- over bilateral strength and an increased BLD in young adults [18]. These findings comply with the well-documented principle of training specificity [19]. However, the aforementioned studies focused on resistance training only. Evidence on different types of neuromuscular training (i.e., heavy-resistance strength training vs. balance training), especially in old adults is missing. This void in the literature is of particular interest because balance training, similar to resistance training, is known to positively affect maximal isometric force (MIF) and rate of force development (RFD) in young, middle-aged, and old adults [20,21]. Although resistance and balance training have the potential to improve force production in old adults, training-induced gains in bilateral MIF following resistance training are more pronounced compared to balance training [22,23]. Resistance training for the lower limbs is mostly performed bilaterally [22], while balance training includes exercise progressions ranging from bilateral to unilateral exercises (i.e., bipedal to unilateral stance) to increase task difficulty [24].

Hence, the aim of this study was twofold. First, we examined age-related differences in BLD and MIF. Second, we evaluated training-induced effects on uni- and bilateral MIF of the leg extensors as well as on BLD. For the latter, we applied different types of training (i.e., bilateral heavy-resistance strength training vs. predominantly unilateral balance training). We hypothesized that a) uni- and bilateral MIF is lower but BLD is higher in old compared to young adults, b) bilateral heavy-resistance strength training and predominately unilateral balance training diversely affect uni- and bilateral MIF as well as the BLD in old adults. Heavy-resistance strength training increases bilateral MIF and reduces BLD after training due to its bilateral characteristics, while balance training increases unilateral MIF and BLD due to its predominately unilateral nature in old adults.

## Methods

### Ethics statement

The Human Ethics Committee at the University of Potsdam approved the study protocol (reference number: 34/2012). Before the start of the study, each participant read, concurred, and signed a written informed consent. All procedures were conducted according to the Declaration of Helsinki.

### Participants

Fifty three old male adults (60–80 years) and 14 young male adults (20–30 years) participated in this study. Their characteristics are summarized in Table 1. Participants were healthy with no history of muscular, neurological, cardiovascular, and metabolic, diseases. Participants’ physical activity was assessed using a self-reported questionnaire that included overall physical activity during a normal week, everyday physical activity (duration, frequency, type), sports activity in and outside of organized clubs (duration, frequency, intensity, type, seasonality) [25]. None of them had previously participated in systematic resistance or balance training.

**Table 1:** Participants’ characteristics.

Characteristic	HRT	BAL	CON	YA
	(*n* = 19)	(*n* = 14)	(*n* = 20)	(*n* = 14)
Age [years]	66.4 ± 4.9	66.3 ± 5.3	66.7 ± 4.0	27.2 ± 3.3
Body Height [cm]	176.6 ± 5.9	173.5 ± 4.7	175.3 ± 5.2	178.6 ± 6.3
Body Mass [kg]	76.6 ± 11.9	77.6 ± 5.9	78.8 ± 9.9	74.6 ± 6.2
BMI [kg/m²]	24.5 ± 2.8	25.8 ± 2.2	25.6 ± 3.0	23.4 ± 1.2
Physical activity [h/week]	10.4 ± 5.5	11.1 ± 6.3	10.8 ± 5.7	13.3 ± 7.6

BMI = body-mass-index, HRT = bilateral heavy-resistance strength training, BAL = predominately unilateral balance training, CON = old adults control group, YA = young adults control group. Physical activity includes leisure time activity (i.e., walking, shopping, gardening) and sports club participation.

### Training programs

The old participants were randomly assigned to 3 experimental groups, (1) bilateral heavy-resistance strength training (HRT: *n* = 19); (2) predominately unilateral balance training (BAL: *n* = 14); or (3) an old adults control group (CON: *n* = 20). Additionally, young adults (YA: *n* = 14) were enrolled in this study to conduct a baseline cross-sectional comparison between young and old subjects and to examine training-induced performance changes of old compared to the general performance levels of YA. YA and CON did not receive training and were asked to follow their regular routines over the course of the study. Subjects of the HRT and BAL group participated in a 13-week training program including 3 sessions a week. The first week constituted a pre-training period where participants were familiarized with the weight training machines and training intensities. Training was performed every other day to provide sufficient resting periods between training sessions. Each session lasted 60 minutes including a 10 minute warm-up on a bicycle ergometer. The HRT group performed bilateral lower limb resistance training at 80% of their one repetition maximum including leg press, leg-extension, calf raise, and foot dorsi-flexor exercises. Subjects performed 3 sets of 10 repetitions for each exercise. Between sets, subjects rested for 2 minutes. Training intensity was individually examined on a weekly basis and training loads were adjusted accordingly.

Balance training was conducted on wobble boards (Togu, Prien-Bachham, Germany), soft mats (Airex, Aalen, Germany) and uneven surfaces (i.e., stability discs). Again, the first week constituted a pre-training period in which participants were familiarized with the exercises and training intensities. Intensity was controlled and adjusted by reducing the base of support (from two- to one-legged stance) and by incorporating unstable surfaces. During the course of the first 2 weeks, task difficulty was increased from standing on two legs to one-legged stance on stable surfaces. During weeks 3 and 4, subjects performed balancing tasks in one-legged stance with a knee flexion angle of 30° on stable surface. During weeks 5–9, participants conducted one-legged balancing tasks on uneven and unstable surfaces. Subjects were allowed to hold on to a nearby wall to provide additional support. The last 3 weeks of training involved one-legged balancing tasks on uneven surfaces with eyes open and hands placed on the hip to remove wall support. Subjects conducted 4 sets of 20 seconds on each surface and in each position, both legs were alternately exercised [26]. Subjects rested for 20 seconds between sets. Training sessions in the HRT- and the BAL-group ended with a 10 minute cool down period on a bicycle ergometer. Every training session was documented and supervised.

### Data registration

Subjects’ MIF was assessed on a leg-press, with each foot resting on a one-dimensional force plate (Kistler Instruments GmbH, Winterthur, Germany). Subjects were positioned on the sledge of the leg-press with hip and knee angles adjusted at 90°. Their waist was fixed and subjects were allowed to stabilize their upper body by holding on to handles attached to the leg-press. Subjects were instructed to avoid exhaling on exertion during maximal efforts. Prior to testing, subjects were familiarized with the testing procedures by performing a warm-up period consisting of 3–5 submaximal isometric contractions. Subsequently, each participant performed 3–4 maximal isometric leg extensor actions for each test condition (i.e., bilateral [BL], unilateral right [ULR], and unilateral left [ULL]). Subjects started with bilateral contractions followed by the unilateral contractions that were randomly presented. They were instructed to perform the exercise as vigorous and fast as possible. The force applied to the force platform was registered at 500 Hz. For subsequent analysis, the signal was filtered by a digital 4^th^ order recursive Butterworth low-pass filter using a cutoff frequency of 50 Hz. MIF was defined as the maximal voluntary force value of the force-time curve, determined under isometric condition. We registered MIF for the BL, ULR, and ULL conditions. BLD was calculated according to the following formula introduced by Howard and Enoka [4]:$BLD[\%]=\left(\left(\frac{MIF(BL)}{MIF(ULR)+MIF(ULL)}\right)-1\right)x100$.

BLDs > 0 indicate a higher bilateral performance compared to the sum of the 2 unilateral performances (i.e., bilateral facilitation). Conversely, a BLD < 0 indicates that the bilateral performance was less than the combined unilateral values (i.e., bilateral inhibition) [4].

### Statistical analyses

Data are presented as group mean values ± standard deviations (SD). Analyses of variance (ANOVA) were used to detect differences between study groups (HRT, BAL, CON, YA) in all variables at pre- and post-tests. Measures of uni- and bilateral MIF as well as BLD were analyzed in separate 3 (Groups: HRT, BAL, CON) × 2 (Time: pre, post) analysis of variance (ANOVA) with repeated measures on Time. Post-hoc tests with Bonferroni-adjusted α were conducted to identify comparisons that were statistically significant. Effect sizes were determined by calculating Cohen’s *d* values [27]. Cohen’s *d* describes the effectiveness of a treatment and determines whether a statistically significant difference is a difference of practical concern. Cohen’s *d* values are classified as small (0.00 ≤ *d* ≤ 0.49), medium (0.50 ≤ *d* ≤ 0.79), and large effects (*d* ≥ 0.8) [27]. To assess correlations between participant’s training-induced changes in BLD and their bilateral MIF, we subtracted the values before and after the training intervention for BLD and bilateral MIF, yielding ∆BLD and ∆MIF-BL separately for HRT and BAL. Correlations between ∆BLD and ∆MIF-BL were assessed using Pearson’s correlation coefficient. Associations are reported by their correlation coefficient *r* and their level of significance. An a priori power analysis [28] with an assumed type I error of 0.05 and a type II error rate of 0.20 (i.e., 80% statistical power) was conducted for measures of MIF and BLD, using an effect size of *f* = 0.40 [17]. Our power analysis revealed that 15 persons per group would be sufficient to achieve significant interaction effects. All analyses were calculated using Statistical Package for Social Sciences (SPSS) version 22.0 (IBM Corp., New York, USA) and significance levels were set at α = 5%.

## Results

Participants of this study can be classified as physically active. All subjects received treatment as allocated. Fifty-three participants completed the 2 training programs and none reported any training-related injuries. Attendance rates amounted to 89% for the HRT-group and 91% for the BAL-group. Table 2 displays means and SDs for all analyzed variables. ANOVA outcomes are shown in Table 3. Overall, there were no statistically significant differences in pre-training values between the 3 old experimental groups.

**Table 2:** Means and SD of outcome measures for each group before and after the intervention period.

	YA	HRT		BAL		CON	
	Pre	Pre	Post	Pre	Post	Pre	Post
BLD [%]	-3.9 (5.9)	-18.7 (6.2)	-5.1 (6.8)	-11.9 (5.9)	-7.2 (5.5)	-19.3 (11.3)	-17.4 (7.3)
MIF-ULL [N]	1223 (167)	784 (168)	860 (159)	788 (74)	833 (102)	824 (197)	768 (168)
MIF-ULR [N]	1263 (207)	807 (164)	870 (173)	808 (137)	873 (111)	848 (220)	802 (183)
MIF-BL [N]	2389 (386)	1294 (256)	1641 (306)	1371 (197)	1565 (194)	1348 (340)	1293 (288)

Note: values are means and standard deviations (SD) in bracket. HRT = bilateral heavy-resistance strength training, BAL = predominately unilateral balance training, CON = old adults control group, YA = young adults control group, BLD = bilateral deficit, MIF-BL = maximal voluntary isometric force of both legs, MIF-ULL = maximal voluntary isometric force of the left leg, MIF-ULR = maximal voluntary isometric force of the right leg

**Table 3:** ANOVA outcome.

	Group	Time	Group × Time
	*F*_(2, 50)_, *p-value (d)*	*F*_(1, 50)_, *p-value (d)*	*F*_(2, 50)_, *p-value (d)*
BLD [%]	*F =* 6.4, *p* <. 001 (1.6)	*F* = 53.8, *p* <. 001 (1.7)	*F* = 15.2, *p* <. 001 (1.9)
MIF-ULL [N]	*F* = 0.1, p >. 05 (0.2)	*F* = 4.3, p <. 05 (0.6)	*F* = 16.3, p <. 001 (1.6)
MIF-ULR [N]	*F* = 0.1, p >. 05 (0.1)	*F* = 5.3, p <. 05 (0.7)	*F* = 10.6, *p* <. 001 (1.3)
MIF-BL [N]	*F* = 1.9, p >. 05 (0.5)	*F* = 70.9, p <. 001 (2.4)	*F* = 42.2, *p* <. 001 (2.8)

Note: HRT = bilateral heavy-resistance strength training, BAL = predominately unilateral balance training, CON = old adults control group, YA = young adults control group, BLD = bilateral deficit, MIF-BL = maximal voluntary isometric force of both legs, MIF-ULL = maximal voluntary isometric force of the left leg, MIF-ULR = maximal voluntary isometric force of the right leg

### Unilateral / bilateral MIF and BLD at baseline

**Fig 1:**
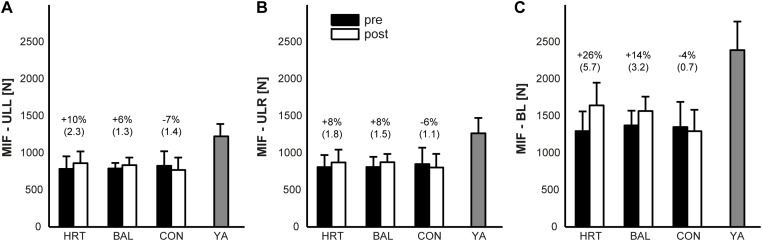
Means of subjects’ strength performance measures during pre and post training testing. A: maximal voluntary isometric force of the left leg (MIF-ULL); B: maximal voluntary isometric force of the right leg (MIF-ULR); C: maximal voluntary isometric force of both legs (MIF-BL). HRT = bilateral heavy-resistance strength training group, BAL = predominately unilateral balance training group, CON = old adults control group, YA = young adults control group. Error bars represent standard deviation (SD); brackets show Cohen’s *d*.

**Fig 2:**
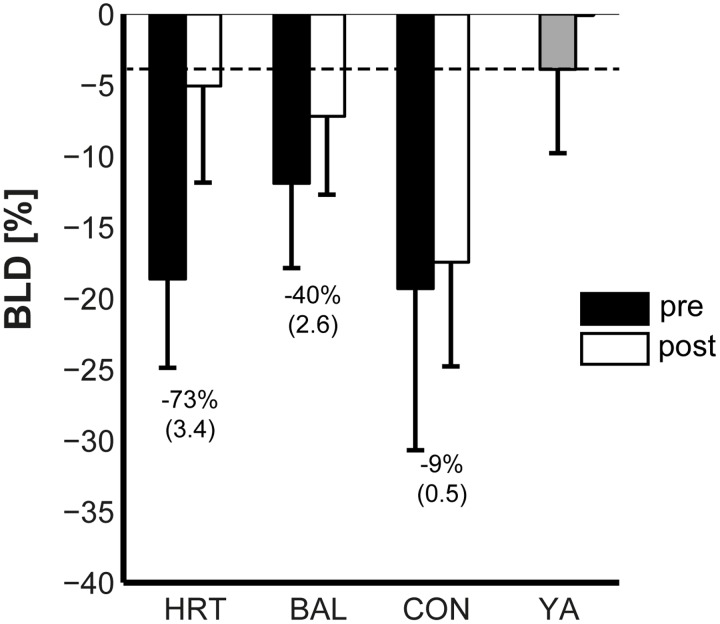
Means of subjects’ bilateral deficit (BLD) in maximum voluntary isometric force during pre and post training testing. HRT = bilateral heavy-resistance strength training group, BAL = predominately unilateral balance training group, CON = old adults control group, YA = young adults control group. Error bars represent standard deviation (SD). Brackets show Cohen’s *d*. The dashed line indicates the BLD of YA.

Subjects’ uni- and bilateral MIF is displayed in Fig. 1 and their BLD in Fig. 2. MIF of the YA-group was significantly higher at baseline (*d* = 2.6–3.4; all p <. 001) compared to the 3 old groups in uni- and bilateral condition. Similar findings were observed for subjects’ BLD. BLD of the YA-group was significantly lower at pre-test (*d* = 2.04; p <. 001) compared to the 3 groups of old adults. No significant differences were present at baseline (all *p* >. 05, *d* = 0.2–0.5) between the 3 old adults groups in MIF-ULL, MIF-ULR, MIF-BL, and BLD.

### Training-induced effects on uni- and bilateral MIF

Compared to the pre-test values, age-related differences were smaller in uni- and bilateral MIF after training (*d* = 0.6–2.4; all p <. 05). Our statistical analyses indicated significant main effects of Time for MIF-ULR (*d* = 0.7; p <. 05), MIF-ULL (*d* = 0.6; p <. 05), and MIF-BL (*d* = 2.4; p <. 001) but not for Group (d = 0.1–0.5; all p >. 05). In addition, significant Group × Time interactions were found for MIF-ULL, MIF-ULR, and MIF-BL (*d* = 1.3–2.8; all p <. 001). Post-hoc analyses revealed significant increases for the HRT-group (MIF-ULL: 10%, *p* <. 001, *d* = 2.3; MIF-ULR: 8%, *p* <. 05, *d* = 1.8; MIF-BL: 26%, *p* <. 001, *d* = 5.7) and the BAL-group (MIF-ULL: 6%, *p* >. 05, *d* = 1.3; MIF-ULR: 8%, *p* >. 05, *d* = 1.5; MIF-BL: 14%, *p* <. 001, *d* = 3.2) as compared to the CON-group (MIF-ULL: -7%, *p* <. 05, *d* = 1.4; MIF-ULR: -6%, *p* >. 05, *d* = 1.1; MIF-BL: -4%, *p* >. 05, *d* = 0.7) for most of the MIF measures. After training, MIF-ULR was significantly higher for HRT and BAL compared to CON (both *p* <. 05, *d* = 0.4–0.5), but similar in both training groups (*p* >. 05, *d* = 0.1). Similar findings were observed for MIF-ULL and MIF-BL.

### Training-induced effects on BLD

After training, the observed age-related difference in BLD was smaller at post-test (p >. 05, *d* = 0.6) compared to pre-test. Our analyses indicated a significant main effect of Group (*p* <. 01, *d* = 1.6) and Time (*p* <. 01, *d* = 1.7). In addition, a significant Group × Time interaction was found (*p* <. 001, *d* = 1.6). Post-hoc analyses revealed significant decreases in the HRT-group (73%, *p* <. 001, *d* = 3.4) and the BAL-group (40%, *p* <. 05, *d* = 2.6) as compared to the CON-group (9%, *p* >. 05, *d* = 0.5). Also, the BLD in HRT and BAL was significantly lower as compared to CON (both *p* <. 001, *d* = 1.5–1.8) but similar in both intervention groups (*p* >.05, *d* = 0.4).

**Fig 3:**
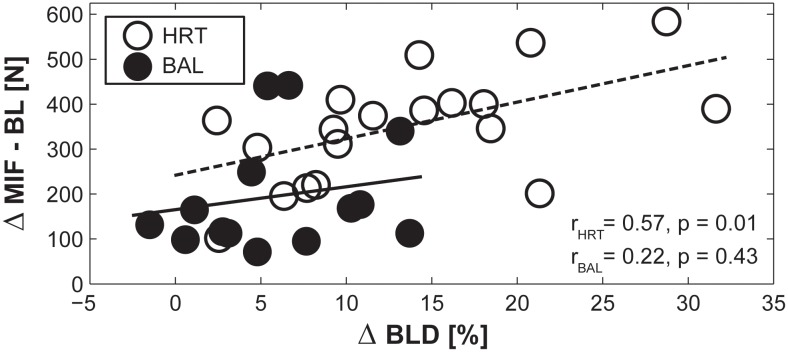
Association between the training-induced changes in maximal voluntary contraction for both legs (∆MIF-BL) and the change in bilateral deficit (∆BLD) for the bilateral heavy-resistance training group (HRT) and the predominately unilateral balance training group (BAL). Pearson’s correlation coefficients (*r*) are displayed separately for HRT and BAL.

Pearson’s correlation coefficients indicated significant and medium-sized associations between ∆BLD and ∆MIF-BL for the HRT-group only. This indicates that participants with large training-induced performance improvements in MIF-BL showed a large reduction in BLD (*r* = 0.57, *p* = 0.01). Of note, no significant correlations were found for the BAL-group (*r* = 0.22, *p* = 0.43; Fig. 3).

## Discussion

To our knowledge, this is the first randomized controlled trial that investigated effects of bilateral heavy-resistance strength training compared to predominately unilateral balance training on measures of uni- and bilateral MIF and BLD in healthy old adults. In addition, findings from old adults were compared to a young adults control group. Our main results can be summarized as follows: (1) at baseline, uni- and bilateral MIF were significantly lower and BLD significantly higher in old compared to young adults. This is in accordance with our first hypothesis; (2) both older intervention groups significantly improved their uni- and bilateral MIF as well as their BLD (HRT-group more than BAL-group); (3) following training, uni- and bilateral MIF and BLD of old adults approximated those of young adults. Thus, our second hypothesis was only partly confirmed.

### Findings on uni- and bilateral MIF and BLD at baseline

Our findings on age-related differences in MIF and BLD are in line with previous studies showing that maximum force production decreases with advancing age [6,14]. Compared to young adults, seniors in the HRT and BAL groups produced 42% (HRT) and 45% (BAL) lower MIF in the bilateral condition. In the unilateral condition, the HRT and BAL groups produced 36% lower MIF in each leg (ULL and ULR) before training. These findings are in a similar range compared to previously published studies which found an age-related force decline of 45% during leg extension [14]. Decreased neuromuscular activity of the agonist muscle, smaller contractile muscle mass (i.e., decrease in cross-sectional area) [29], and changes in muscle architecture [30] have been identified to contribute to age-related deficits in muscle strength. Regarding BLD, previous findings on age-related differences in BLD were ambiguous. In the present study, BLD in old adults (HRT: 19%, BAL: 12%, and CTL: 19%) was significantly higher at baseline compared to that of young adults (4%). This indicates that old adults were less capable of generating muscular force during synchronous bilateral movements. Lower neuromuscular activation during bilateral compared to unilateral contractions has been discussed as one possible agent to explain BLD in old adults [31]. A prominent mechanism for higher BLD in old compared to young subjects is the inhibition of transcallosal pathways during bilateral contractions [10]. Bilateral contractions appear to afford higher cortical activations and the recruitment of more brain regions compared to unilateral contractions [32]. Thus, more brain activity seems to be required to coordinate bilateral contractions. However, the recruitment of additional brain regions and the possibilities to increase brain activity seems to be limited in older compared to young adults [33].

### Effects of heavy-resistance training on uni- and bilateral MIF and BLD

Following bilateral heavy-resistance training, the observed age-related differences between young and old adults in uni- and bilateral MIF at baseline were mitigated. Compared to young adults, seniors reduced their age-related deficit by 11% in bilateral MIF (HRT and BAL), by 4% (HRT and BAL) in unilateral MIF of the right leg, and by 7% (HRT) and 4% (BAL) in unilateral MIF of the left leg after the intervention period. Thus, resistance training is a suitable approach to improve muscle strength [34]. In fact, heavy-resistance training resulted in significant improvements in maximum voluntary contraction and RFD (cf. [35] for an overview). In an early study, Frontera and colleagues [36] were able to show that 12 weeks of resistance training (3 times/week) at 80% of the one repetition maximum led to improved maximal knee extensor/flexor strength in old adults. In another study, old adults showed gains in MIF and RFD of the leg extensors after 13 weeks of resistance training (3 times/week, 80% of one repetition maximum) [22]. Several underlying neuromuscular factors were identified to explain training-induced gains in muscle strength in old adults [35–37]. Namely, an increase in muscle mass, predominantly by increased muscle cross-sectional area [38]. Further, enhanced neuromuscular activation (i.e., improved recruitment patterns, discharge rates, and synchronization of motor units), improved synergistic muscle activation, and a reduced co-activation of antagonistic muscles [35].

In addition, the present study revealed that bilateral heavy-resistance training improved subjects’ BLD. In fact, following heavy-resistance training, BLD was predominately reduced by improved bilateral MIF. Further, increases in bilateral MIF were positively correlated to a decreased BLD after training (cf. Fig. 3). Our findings are in line with the results of a previous study that revealed larger effects of bilateral resistance training on BLD as compared to unilateral resistance training in old adults [17]. After 26 weeks of bilateral training (2 times/week) BLD of the knee extensors was reduced whereas unilateral training (2 times/week) had only minimal effects on BLD.

What is the underlying mechanism that results in improved BLD following bilateral resistance training? Bilateral heavy-resistance training might condition the brain to increase cortical activation and to recruit more brain regions in both hemispheres when performing bilateral contractions [32], thereby reducing cortical inhibition [12]. In fact, following short-term resistance training (i.e., 2 weeks, 3 sessions/week), cortical inhibition was reduced in old adults [39]. Furthermore, increased cortical activity and the recruitment of additional brain regions have been shown during bilateral compared to unilateral contractions [32]. Thus, resistance training might enhance the activation of more brain regions during bilateral muscle contractions, leading to increased muscular output triggered through direct cortico-spinal pathways [40].

### Effects of balance training on uni- and bilateral MIF and BLD

Participants’ MIF significantly improved after predominately unilateral balance training. In previous research, it has been shown that MIF of the knee flexors and extensors improved following 12 weeks of balance training (2 times/week) in adults aged >65 years [41]. Also, old adults’ MIF and RFD of the leg extensor increased after 13 weeks of balance training (3 times/week) [23]. Our study is in line with these findings. Balance training induced increases in MIF and RFD can be explained by a study from Hortobagyi and colleagues [42]. Findings indicated that old adults perform strength-related everyday-tasks (i.e., ascend/descend of stairs, rising from a chair) near their maximal capacity. Balance training involves standing on one leg for 20–40 s [43]. While this seems trivial, the relative load might be close to the maximal capacity of old adults. Thus, balance training not only challenges postural control but also muscle strength when the loading is appropriate.

Different mechanisms appear to be responsible for the observed findings. Increases in motoneuron firing frequency are a prominent agent for training-induced improvements in force-generating capacity following balance training [21]. Further, it has been reported that balance training results in lower presynaptic inhibition and thus larger motor output during force generating tasks [20]. On the cortical level, adaptations to balance training seem to be highly task-specific [44,45]. More precisely, following balance training, cortical excitability was reduced during the execution of a postural task [44]. However, when balance-trained subjects were measured in a strength task (i.e., dorso-flexion of the ankle), enhanced cortical excitability was evident. It was argued that improved force production following balance training might additionally be caused by an enhanced direct cortical drive to the agonistic muscles [46].

Contrary to our expectations, the BAL-group also reduced BLD. Older adults in the BAL-group (BLD of 7%) almost reached the level of young adults (BLD of 4%) after training. One can argue that the improvements described for MIF following balance training may also affect BLD. Our study revealed larger training-induced increases in bilateral MIF (14%, d = 3.2) as compared to unilateral MIF (ULL: 6%, d = 1.3; ULR: 8%, d = 1.5), thereby decreasing the BLD. Following balance training, reduced cortical excitability has been shown during the performance of a balance tasks and increased cortical excitability during the execution of an explosive strength task [44,45]. Even though speculative, it appears plausible to argue that balance training modulates cortical excitability by facilitating the reduction in BLD [32]. Of note, predominately unilateral balance training seems to have bilateral benefits that might be sufficient to significantly reduce BLD. Additionally, Taube [20] argues that balance training and the accompanied improvement in postural control lead to adaptations in subcortical structures of the brain (i.e., basal ganglia and cerebellum). For example, activity in subcortical brain regions increased with increasing task automatization [47] and following 6 weeks of balance training [48]. Following this argument, balance training might induce a shift in activation from cortical to subcortical areas that bypasses interhemispheric inhibition of the motor cortices during bilateral contractions. However, further research is needed to experimentally examine the described mechanisms since our data do not allow us to directly attribute the improvements after balance training to specific parts of the cortical system.

## Conclusions

In conclusion, our findings demonstrate that old adults show decreased levels of uni- and bilateral muscular strength and increased levels of BLD compared to young adults. Interestingly, both uni- and bilateral MIF and the magnitude of BLD can be positively influenced by bilateral heavy-resistance strength training and by predominately unilateral balance training. After training, both intervention groups almost reached the MIF and BLD levels of young adults. This suggests that bilateral heavy-resistance training as well as predominately unilateral balance training interventions can be used to improve uni- and bilateral MIF and to reduce BLD in older adults.
